# Cardiac toxicities in multiple myeloma: an updated and a deeper look into the effect of different medications and novel therapies

**DOI:** 10.1038/s41408-023-00849-z

**Published:** 2023-05-19

**Authors:** Jean El-Cheikh, Nour Moukalled, Florent Malard, Ali Bazarbachi, Mohamad Mohty

**Affiliations:** 1 Division of Hematology and Oncology, Department of Internal Medicine, American University of Beirut Medical Center, Beirut, Lebanon; 2 Department of Hematology, Saint Antoine Hospital, AP-HP, Paris, France; 3 Sorbonne University, INSERM UMRs 938, Centre de Recherche Saint-Antoine (CRSA), Paris, France

**Keywords:** Myeloma, Risk factors

## Abstract

With the continuous improvement in survival of cancer patients, including those with multiple myeloma, related to the novel treatment agents and therapeutic approaches, the probability for patients to develop cardiovascular disease has significantly increased, especially in elderly patients and those with additional risk factors. Multiple myeloma is indeed a disease of the elderly population and so these patients are, solely by age, at an increased risk of cardiovascular disease. Risk factors for these events can be patient-, disease- and/or therapy-related, and they have been shown to adversely impact survival. Cardiovascular events affect around 7.5% of patients with multiple myeloma and the risk for different toxicities has considerably varied across trials depending on patients’ characteristics and treatment utilized. High grade cardiac toxicity has been reported with immunomodulatory drugs (odds ratio [OR] around 2), proteasome inhibitors (OR 1.67–2.68 depending on the specific agent, and generally higher with carfilzomib), as well as other agents. Cardiac arrhythmias have also been reported with various therapies and drug interaction plays a significant role in that setting. Comprehensive cardiac evaluation before, during and after various anti-myeloma therapy is recommended and the incorporation of surveillance strategies allows early detection and management resulting in improved outcomes of these patients. Multidisciplinary interaction including hematologists and cardio-oncologists is critical for optimal patient care.

## Introduction

Ischemic heart disease and stroke are the two leading noncommunicable causes of death and disability on a global scale, the former being responsible for 16% of the world’s total deaths in 2019 [1]. When we zoom in on what happens in Europe, ischemic heart disease is, for men and women, the most frequent cause of death with a rate of 1 in 5 [2], while for stroke, the rate is 1 in 10. Although cancer is the leading cause of death in some European countries, it is cardiovascular (CV) disease that remains the most common cause of death in the majority of these countries [3]. Cardiovascular diseases are frequent; cancer is frequent, and several anticancer treatments can additionally cause CV toxicities. With the continuous improvement in survival of cancer patients related to the novel treatment agents and therapeutic approaches, the probability for these patients of developing CV disease has significantly increased, especially in elderly patients and those with additional risk factors [4].

The general CV risk factors include age, family history of CV disease, arterial hypertension, in addition to modifiable risk factors such as diabetes, hypercholesterolemia, smoking, high alcohol intake, obesity, and sedentary lifestyle. The common CV complications of cancer treatment include myocardial dysfunction and heart failure (HF), in addition to coronary artery disease (CAD), followed by valvular heart disease and arrhythmias including prolongation of the QT interval, in addition to arterial hypertension, thromboembolic disease and other causes of peripheral vascular disease or stroke, pulmonary hypertension and very rarely, pericardial disease [4].

Multiple myeloma (MM) is indeed a disease of the elderly population who are, solely by age, at an increased risk of CV disease [5, 6]. Cardiovascular events might affect up to 7.5% of individuals with MM, due to a combination of patient-, disease- and therapy-related factors, adversely impacting survival [7]. Patient related factors include age, hypertension, diabetes mellitus, ethnicity, and male gender, among others. In addition, we have extensive knowledge about the significance of renal impairment and anemia, as important risk factors, both of which are more commonly seen among patients with MM. Disease related factors can also include the presence of increased levels of immunoglobulins which is associated with hyper-viscosity; as well as the possible cardiac amyloid deposition in those with AL amyloidosis [7]. Moreover, there are therapy-related factors (cardiotoxic chemotherapy, corticosteroids, proteasome inhibitors (PIs), immunomodulatory drugs (IMiDs), and radiotherapy, in addition to the growing knowledge regarding the possible cardiotoxicity associated with other novel agents). A large study that included 1723 MM patients who were under treatment with corticosteroids and ≥3 drugs (bortezomib, immunomodulatory derivatives, and alkylating agents or anthracyclines) provided the first known comparison of cardiac event risk in patients with MM versus 8615 (a group five times as large), age- and gender-matched patients without MM. The cardiac event risk was greater in MM patients for any cardiac event (60.1% versus [vs.] 54.6%), dysrhythmias (29.1% vs. 13.1%), cardiomyopathy (5.4% vs. 2.0%), and more precisely, for congestive HF (15.1% vs. 5.2%), compared with those without MM [8]. The incidence of hypertensive or arterial events and ischemic heart disease was however similar between the 2 groups [8]. Anti-myeloma drugs can also be associated with an array of cardiac toxicities including HF, arrhythmias such as bradycardia or atrioventricular block (bortezomib, cyclophosphamide, thalidomide), atrial fibrillation (cyclophosphamide, melphalan), supraventricular tachycardias (cyclophosphamide, melphalan, bortezomib), and ventricular tachycardia/fibrillation (cyclophosphamide, bortezomib, carfilzomib) [4]. We herein present an extensive literature search to provide an updated review regarding cardiac complications in the landscape of modern MM management, utilizing a deeper look into the different classes of antimyeloma therapies.

## Conventional chemotherapy

At present, there is still some limited use of anthracyclines (doxorubicin), e.g., as in the old regimen of vincristine-doxorubicin [adriamycin]-dexamethasone (VAD), or the combination of bortezomib–doxorubicin–dexamethasone in the PAD regimen [9], which carries a risk of left ventricular dysfunction (VD) that can vary between 3 and 50%. Moreover, alkylating agents such as cyclophosphamide, which is still widely used in the management of MM, might induce a variable risk of VD ranging between 7 and 28% [4]. Previously, Sunami et al. had reported results from a phase I/II study of sequential VAD, high dose cyclophosphamide utilized for stem cell mobilization, followed by tandem autologous stem cell transplant (ASCT) using melphalan conditioning in a total of 40 patients, noting only 2 unspecified grade III CV toxicity events during induction with VAD, and 1 event post first transplant but none during high dose cyclophosphamide, or the second tandem transplant [10]. Similarly, the intensive chemotherapy regimen CEVAD (cyclophosphamide, etoposide, vincristine, Adriamycin, dexamethasone) was associated with around 5.5% cardiotoxicity (2 out of 36 patients, both of whom were previously treated with high doses of adriamycin) [11]. Autologous stem cell transplant using high dose melphalan still plays an important role in the management of newly diagnosed and relapsed disease. Atrial fibrillation (AF) and supraventricular arrhythmias have been reported with melphalan [12, 13], while there have been few reports about ventricular arrhythmias with this agent [14]. Long-term follow up of 12 patients with MM post tandem ASCT, in the era preceding the availability of novel therapies, had shown early post-transplant toxicities including diastolic dysfunction, increased left atrial pressure, and functional mitral regurgitation, which were clinically silent and reversible upon follow up over 6 years [15]. A recent retrospective analysis of patients who underwent ASCT with either Melphalan 150 or 200 mg/m^2^ (including around 459 total patients over the period extending between 1995 and 2019), has reported a low incidence of cardiac toxicity (around 2.6%), without specifying the type of toxicity, however noting a slightly higher incidence in the 150 mg/m^2^ group, possibly related to pre-transplant underlying comorbidities [16].

The use of high dose chemotherapy including combination regimens such as dexamethasone, cyclophosphamide, etoposide, and cisplatin (DCEP), bortezomib-DCEP (VDCEP); as well as bendamustine are generally limited to the refractory setting where patient’s performance status and comorbidities would further increase the risk for cardiac toxicity, and thus require special attention and management.

## Immunomodulatory drugs (IMiDs)

Some of the IMiDs have been associated with cardiotoxicity, in addition to their well-known increased risk of vascular complications including venous thromboembolism among others. The underlying mechanism of this cardiotoxicity is yet to be unraveled, however proteasome-mediated protein degradation related to the effects of binding to cereblon, a part of E3 ubiquitin ligase, leading to its activation; as well as endothelial injury and dysfunction have been suggested to play an important role [17].

Thalidomide is nowadays less widely used on a global scale, but higher doses of this drug in particular can be associated with bradycardia and atrioventricular conduction abnormalities [18]. A meta-analysis of 11 phase 3 randomised controlled trials (RCTs) reported on cardiotoxicity in MM patients (newly diagnosed and/or relapsed), treated with IMiDs and/or PIs. This analysis showed an odds ratio (OR) of 2.05 (95% confidence interval [CI] 1.30–3.26) for high grade (grade ≥3) Common Terminology Criteria for Adverse Events (CTCAE)- cardiotoxic events (747 out of 2733) in myeloma patients treated with IMiDs (specifically thalidomide or lenalidomide), as compared to those not receiving IMiDs (545 out of 2727), however, there may have been a bias due to the confounder of high-dose dexamethasone which was used in the early days in combination with thalidomide and lenalidomide [19]. Lenalidomide in combination with dexamethasone has also been associated with an increased risk of myocardial infarction (MI) when compared to dexamethasone alone (1.98% versus 0.57%), as well as that of stroke (3.4% versus 1.7%) as reported in 2 phase III studies in patients with relapsed disease [20]. Data regarding adverse events (AEs) with Thalidomide compared to all other agents from the Food and Drug Administration (FDA) Adverse Events Reporting System (FAERS) database have shown an OR of 2.5 (2.2544–2.7749; *p* < 0.0001) for AF, 2.77 (2.5856–2.9739; *p* < 0.0001) for cardiac failure, and 1.5 (1.4081–1.6540; *p* < 0.0001) for CAD [21]. For Lenalidomide, the ORs were 2.8 (2.7060–2.9229; *p* < 0.0001), 1.68 (1.6315–1.7480; *p* < 0.0001) and 1.08 (1.0508–1.1306; *p* < 0.0001) respectively [21]. In addition, there have been two case reports of myocarditis possibly related to lenalidomide [22, 23], although causal effect is yet to be proven. Similarly, the KEYNOTE-183 randomised, open-label, phase 3 trial performed at 97 medical centers across 11 countries in MM patients previously treated with at least two lines of therapy, which compared pomalidomide combined with dexamethasone with or without the addition of pembrolizumab, reported death related to MI, cardiac failure, pericardial hemorrhage and myocarditis, the latter being possibly related to pembrolizumab given the well-known safety profile of PD-1 inhibitors [24]. Based on the FAERS database, the OR of AF with pomalidomide was 3.2 (3.0329–3.5223; *p* < 0.0001), that of cardiac failure was 1.3 (1.2001–1.4152; *p* < 0.0001) and that of CAD was 0.65 (0.5950–0.7258; *p* < 0.0001) [21]. Conversely, novel agents such as Iberdomide, a cereblon E3 ligase modulator (CELMoD) has not yet been associated with significantly increased risk for cardiac toxicities.

## Proteasome inhibitors (PIs)

PIs including bortezomib and carfilzomib had a reported risk of VD of 2–25% [4]. These agents had also been evaluated in the previously mentioned meta-analysis [19] with seven RCTs comparing PIs with control, and two RCTs comparing bortezomib against carfilzomib. The OR for all grades of cardiac toxicity was 1.47 (95% CI 1.19–1.82). High-grade cardiotoxicity was more frequent with bortezomib when compared to the control group (OR = 1.67; 95% CI 1.17–2.40), however, the risk of cardiac failure or other cardiac events was much higher with the use of carfilzomib (OR = 2.68; 95% CI 1.63–4.40). In addition, a retrospective analysis of patients from phase II/III studies had shown no significant difference in the incidence of cardiac toxicities with bortezomib, where the incidence of grade 3 HF was 1.2–4.7%, that of grade 3 ischemic heart disease was 0.4–2.7%, that of arrhythmias was 0.6–4.1% and cardiac death was 0–1.4% [25]. Similarly, a retrospective propensity matched study of 1790 patients compared cardiac toxicity with bortezomib (895 patients) to that with lenalidomide (895 patients) and noted no significant difference in the rate of hospitalization related to HF (HR 1.54, 95% CI, 0.84–2.82), that of MI or cardiac procedures [26]. The FAERS database indicated an OR of 4.7 (4.3882–5.1133; *p* < 0.0001), 1.24 (1.1390–1.3608; *p* < 0.0001), and 9.27 (8.7162–9.8734; *p* < 0.0001) respectively for AF, cardiac failure and CAD with bortezomib when compared to all other drugs in the reporting system [21].

The suggested underlying mechanism for cardiac toxicity with bortezomib possibly relates to the impairment of activation of nuclear factor kappa-beta (NF-κB) among other transcription factors which affects angiogenesis and survival of cardiac myocytes, with protein accumulation and mitochondrial dysfunction affecting contractility [27], in addition to the effects on vascular smooth muscle cells which might exacerbate the vulnerability of atherosclerotic plaques [28].

Extensive data is available regarding the cardiac toxicities expected with carfilzomib. A systematic review and meta-analysis of RCTs which had estimated the relative risk of HF with carfilzomib-based regimens in patients with MM, noted an OR of 2.34 (95% CI 1.66–3.32) for all grade and 2.69 (95% CI 1.77–4.09) for high grade cardiotoxicity, respectively [29]. The incidence rate of CV events with carfilzomib treatment has varied across trials. In 2018, an analysis evaluated phase 1–3 trials with >2000 relapsed/refractory (R/R) MM patients exposed to carfilzomib to describe the incidence of CV treatment emergent adverse events (TEAEs) [30]. The rates of any-grade, and grade ≥3 CV TEAEs (cardiac failure, hypertension, dyspnea, and ischemic heart disease) for the phase 3 ASPIRE, ENDEAVOR, and FOCUS trials were evaluated. Looking at cardiac failure firstly, and the trials of ASPIRE and ENDEAVOR with doses of 27 mg/m^2^ and 56 mg/m^2^, respectively, the overall risk of cardiac failure (any grade) varied between 6.4% and 8.2%, respectively. For grade ≥3, the respective rates were 3.8% and 4.8%. Ischemic heart disease was also increased because of carfilzomib ((any grade 5.9% compared to 2.8%) while grade ≥3 was at 3.3% compared to 1.7%). The use of carfilzomib was also associated with an increase in blood pressure (any grade 15.8%). However, although patients receiving carfilzomib had a numeric increase in the rates of any-grade and grade ≥3 cardiac failure, dyspnea, and hypertension, the frequency of discontinuation or death due to these cardiac events was low and comparable between the carfilzomib and control arms. In the ASPIRE trial, the cumulative incidence of MM disease progression or death at 18 months was 35% (carfilzomib) vs. 52% (control). In the ENDEAVOR trial, the respective figures were 48% (carfilzomib) vs. 78% (control). The results, including the overall survival (OS) benefit, showed that the benefit of carfilzomib treatment in terms of reducing progression or death outweighed the risk for developing cardiac failure or hypertension in most patients [30]. An Analysis of the FAERS recently included 19,486 AEs noted with carfilzomib, out of which 14.8% were related to either acute MI, HF (5.4%), arrhythmia (both supraventricular and ventricular), pericardial disease or hypertension (3.4%) [31]. In addition, when compared to all other agents in the FDA reporting system, the OR for cardiac toxicity with carfilzomib was 4.1 (3.6794–4.7870; *p* < 0.0001), 6.98 (6.4820–7.5195; *p* < 0.0001) and 2.16 (1.9343–2.4153; *p* < 0.0001) for AF, cardiac failure and CAD respectively [21].

The potential risk factors for CV adverse events (CVAEs) associated with carfilzomib have not been fully understood. Nonetheless, available data suggest a possible dose-related effect. The first systematic review and meta-analysis (24 studies; 2594 MM patients) of carfilzomib associated CVAEs explored the incidence of these events and compared the rates of carfilzomib related AEs among different doses and companion therapies. The review included all-grade and grades ≥3, and those were seen in 18.1% and 8.2%, respectively. In comparison with doses <45 mg/m^2^, carfilzomib doses of ≥45 mg/m^2^ were associated with increased high-grade CVAEs (6.4% vs. 11.9%, respectively; *p* = 0.02). Median age >65 years, prior myeloma therapies, and concurrent myeloma therapies were associated with higher CVAEs [32]. In a prespecified interim analysis of the randomized, open-label, phase 3 ARROW trial (https://clinicaltrials.gov/show/NCT02412878), Moreau et al., compared patients randomly assigned (1:1) to receive carfilzomib once a week (70 mg/m^2^, *n* = 238) or twice a week (27 mg/m^2^, *n* = 235), both with dexamethasone. There was no significant difference in cardiac failure or ischemic heart disease between the two dosing regimens [33].

Regarding the possible underlying pathogenesis of cardiotoxicity associated with carfilzomib, it appears that there is a detrimental effect on the mitochondria whereby carfilzomib treatment reduces the mitochondrial membrane potential, ATP production, and mitochondrial oxidative respiration, increasing the mitochondrial oxidative stress. This finally results in decreased contractility of cardiomyocytes. In addition, there is also an indication that carfilzomib treatment downregulates the expression of genes involved in extracellular matrices, integrin complex, cardiac contraction, as well as autophagy, and upregulates stress responsive proteins including heat shock proteins [34, 35]. In addition, there have been data indicating a possible role for the pyruvate oxidation pathway associated with mitochondrial dysfunction as evidenced by the down-regulation of pyruvate and up-regulation of lactate dehydrogenase B among patients who experienced CVAEs with carfilzomib [36].

Cardiac complications related to carfilzomib are mostly reversible. Patients have developed severe cardiac failure overnight requiring intensive care but 24 to 48 h later, the symptoms improved and have more or less returned to normal. In the ASPIRE trial, 60% vs. 37.5% of cardiac failures (any-grade) resolved in the carfilzomib vs. control arms, respectively. Figures for the ENDEAVOR and FOCUS trials were 36.8% vs. 61.5%, and 50% vs. 14.3%, respectively. The majority resolved without sequelae [30].

In an effort to identify patients at the highest risk of cardiac complications, a prospective, observational, multi-institutional study (PROTECT: Prospective Observation of Cardiac Safety with Proteasome Inhibitor) attempted to define risk factors and outcomes in patients with MM receiving PIs [37]. This study reported on 95 patients who were treated with either bortezomib (*n* = 30) or carfilzomib (*n* = 65). Monitoring occurred over 18 months for development of CVAEs which were significantly higher with carfilzomib than with bortezomib. Patients with CVAEs had poorer survival. The median OS for patients experiencing a CVAE was 18.1 months (95% CI 11.6-not reached) and for patients not experiencing a CVAE, median OS had not been reached at the time of analysis (*p* < 0.001). On further analysis for predictors of first CVAE, the authors found that carfilzomib-based therapy was associated with a higher risk of CVAEs compared with bortezomib-based therapy (hazard ratio (HR) 3.0; 95% CI 1.1–8.4; *p* = 0.04). Elevated baseline B type-natriuretic peptide (BNP) levels were associated with higher risk of CVAEs (HR 4.1; 95% CI 2.1–8.1; *P* < 0.001) [37]. Most cardiac events occur during the first 1 to 3 months of treatment and in this study, baseline BNP levels that became elevated during the first cycle of treatment were associated with a higher risk of CVAEs compared with persistently normal BNP values (HR 9.5; 95% CI 4.3–20.7; *p* < 0.001). Patients with zero or one baseline traditional CV risk factor had a lower risk of CVAEs (HR 0.5; 95% CI 0.3–0.9; *p* = 0.02). No evidence of increased risk of CVAEs with respect to time from myeloma diagnosis to enrollment in PROTECT was observed (*p* = 0.9) [37].

Ixazomib, a third PI is nowadays utilized for some newly diagnosed and most commonly R/R MM; the risk for cardiotoxicity with ixazomib being more along the lines of bortezomib (OR of 1.56; 95% CI 0.84–2.90), where in the RCTs for patients treated with ixazomib, there was no clear signal of increased cardiac toxicity [19].

Importantly, treatment protocols for multiple myeloma in general depend on at least doublet, triplet (most commonly PI and IMiD), or more recently quadruplet regimens, where the synergistic effects of multiple agents might lead to augmented risk of toxicity (including cardiac events) when such therapies are co-administered together, especially with the expected steroids induced endothelial stress [17]. Drug-drug interaction needs to be carefully considered in patients with MM, who are generally treated with multiple medications including anti-infective drugs and/or antidepressants (specifically for patients who develop neuropathies), which would further increase the risk of several cardiac toxicities including arrhythmias given the possibility for development of QT prolongation.

## Anti-CD38 therapies

The CANDOR study comparing daratumumab, carfilzomib, and dexamethasone (KdD, *n* = 308) with carfilzomib and dexamethasone (Kd, *n* = 153) showed that interestingly, when focussing on cardiac failure, the rate was lower in the KdD arm compared with the Kd arm (*n* = 12 [3.9%] vs. *n* = 13 [8.5%], respectively) [38]. The latter raised the question of the cardioprotective effect from daratumumab. Another smaller study prospectively evaluated 25 patients with R/R MM who received daratumumab in combination with carfilzomib and dexamethasone (KdD, *n* = 14) or Kd as a control (*n* = 11). Patients were followed for a median of 10 months for CVAEs. The two treatment groups did not significantly differ in baseline demographic characteristics (*p* > 0.1 for all). Cardiac function test values were compared. In the KdD group, no significant change in markers of ventricular systolic function was observed. However, these markers deteriorated in the Kd group; left ventricular ejection fraction (LVEF), left ventricular global longitudinal strain (LV GLS), tricuspid annular plane systolic excursion and right ventricular free wall longitudinal strain significantly decreased from baseline to second visit (*p* < 0.05). A significant group interaction (with *p* < 0.05) was observed for the above-mentioned changes. Cardiovascular adverse events occurred more frequently in the Kd than the KdD group (45% vs. 28.6%). KdD was associated with preserved post-treatment cardiac systolic function and lower CVAE rate compared with Kd [39]. The mechanism behind this speculated cardioprotective effect, although not fully understood, could be related to inhibition (by daratumumab) of ectoenzymatic activity on the coenzyme NADP+, however this still needs additional exploration and evaluation for confirmation. Available data suggest development of hypertension in around 5–6% of patients receiving daratumumab.

Isatuximab is an alternative CD38 targeting immunoglobulin utilized in MM. A phase II trial that evaluated isatuximab as monotherapy or combined with dexamethasone reported dyspnea in 17.4% and 14.5%, respectively with no other cardiac toxicities noted [40]. Additionally, the phase 3 IKEMA trial of isatuximab (IsaKd) vs. Kd did not show any difference in cardiac events between the two treatment arms [41]. This study reported grade ≥3 hypertension in 36 patients (20%) and 24 patients (20%) of the 2 groups respectively, while cardiac failure was reported in 13 patients (7%), of which 7 events were grade ≥3, and 8 patients (7%), of which 5 events were ≥3, respectively, and ischemic heart disease in 8 patients (5%) of which 2 events were grade ≥3 compared to 5 patients (4%), of which 2 events were ≥3, respectively [41].

## Other therapies including elotuzumab, bispecific agents and chimeric antigen receptor T cell therapies (CAR-T)

In the ELOQUENT-2 trial, grade 3 or 4 hypertension were reported in 1.3% of those who received Elotuzumab in combination with lenalidomide and dexamethasone as compared to 2.2% in those who received lenalidomide with dexamethasone alone, without additional cardiac toxicities noted [42].

Chimeric Antigen Receptor T cell therapy (CAR-T), currently utilized in the treatment of R/R MM, can lead to direct and indirect cardiac toxicities, mainly through the associated cytokine release syndrome (CRS), where the increase in several inflammatory markers and cytokines can lead to hypotension, tachycardia/arrhythmias partly related to the activation of the sympathetic nervous system leading to cardiac stimulation, in addition to VD related to possible myocyte shortening and mitochondrial dysfunction [43]. No cardiac failure was reported with Idecabtagene vicleucel in the KarMMa trial [44]. Initial as well as updated results for 2-year follow up from the CARTITUDE-1, a phase Ib/II study which evaluated the safety and efficacy of ciltacabtagene autoleucel (cilta-cel) reported hypertension in 6.2% of patients with no additional cardiac toxicities [45, 46], while the indirect implications related to CRS on cardiac function still require additional considerations. Similar implications should be considered with the various bispecific agents that target cluster of differentiation3 (CD3) and either b cell maturation antigen (BCMA) or G-protein coupled receptor family C group 5 member D (GPRC5D) among others, which have also been shown to be associated with increased risk for CRS, with however no reported cardiac failure thus far [47, 48].

**Fig. 1:**
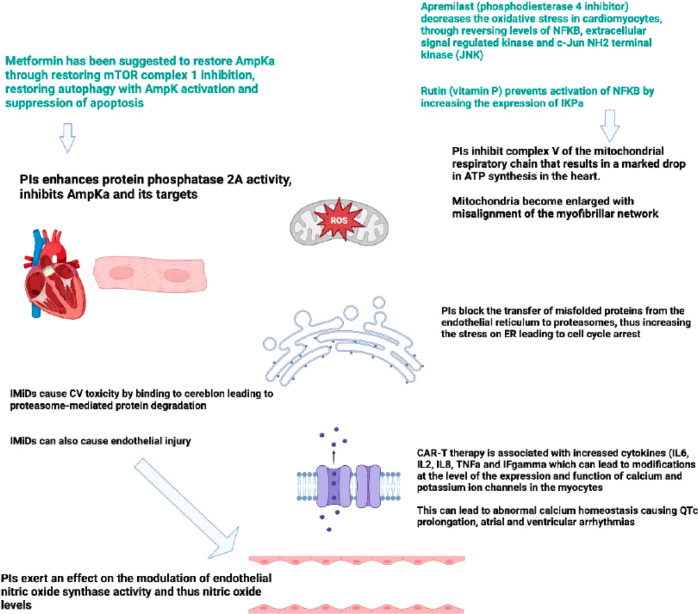
Suggested underlying mechanism of cardiac toxicity with different anti-myeloma treatment agents and few of the suggested preventive/treatment medications. AmpKa activated protein kinase alpha, mTOR mammalian target of rapamycin, PIs proteasome inhibitor, ROS reactive oxygen species, IMiD immunomodulatory agent, CV cardiovascular, NFKB nuclear factor kappa-light-chain enhancer of activated B cells, ATP adenosine triphosphate, ER endothelium reticulum, CAR-T chimeric antigen receptor T-cell therapy, IL interleukin, TNF-a tumor necrosis factor alpha, IF interferon.

Table 1 includes a summary of reported cardiac toxicities in patients with MM across several trials. Figure 1 illustrates the mechanism of cardiac toxicity with different anti-myeloma agents, and the possible protective as well as treatment options for such complications.

**Table 1:** Summary of reported cardiac toxicities in patients with Multiple myeloma.

Author, year	Study, type	Number of patients, median follow up	Multiple myeloma Treatments	Cardiac toxicity	Rate of discontinuation of treatment related to cardiac toxicity %
Stewart et al., 2015 [53]	ASPIRE, phase III RCT	792 (396 each arm), 32.3 months	KRd vs Rd	-All grade with KRd 14.3% hypertension, 6.4% cardiac failure and 5.9% ischemic heart disease Grade ≥ 3: -Cardiac failure 3.8% with KRd and 1.8% with Rd -Hypertension 4.3% with KRd and 1.8% with Rd -Ischemic heart disease 3.3% with KRd and 2.1% with Rd	−0.5% for cardiac failure with KRd and 0.8% with Rd −0.3% for hypertension for each arm −1.3% for ischemic heart disease with KRd and 0.5% with Rd 4 deaths with carfilzomib related to MI or cardiac failure
Dimopoulos et al., 2016 [54]	ENDEAVOR, phase III RCT	929 total, around 11.1–11.9 months	Carfilzomib or bortezomib with dexamethasone	-Hypertension 24.8% with carfilzomib vs 8.7% with bortezomib -Cardiac failure 8.2% with carfilzomib vs 2.8% with bortezomib -Ischemic heart disease 2.6% with carfilzomib vs 1.9% with bortezomib	−2.8% for cardiac failure with carfilzomib vs 0.9% with bortezomib −1.1% for ischemic heart disease with carfilzomib vs 0.7% with bortezomib −0.4% for hypertension with carfilzomib vs 0% with bortezomib
Danhof et al., 2016 [55]	Real life experience	22, NA	Carfilzomib combinations	23% left VD	NA
Kistler et al., 2017 [8]	Retrospective matched controlled	1723, 9 months	corticosteroids and ≥ 3 drugs (bortezomib, immunomodulatory derivatives, and alkylating agents or anthracyclines)	-Any cardiac event HR 2.2 (95% CI 1.9–2.5) -Dysrhythmia HR 4.1 (95% CI 3.5–4.8) -Congestive HF HR 2.9 (95% CI 2.2–3.7) Cardiomyopathy HR 2.6 (95% CI 1.8–3.8) -Conduction disorders HR, 1.7 (95% CI 1.2–2.5)	NA
Chari et al., 2018 [30]	Analysis including 11 RCTs	Total 2044, Variable	Carfilzomib-based	Any grade: -Cardiac failure 6.7% -Hypertension 18.5%	Around 3.7% total (1.9% for cardiac failure and 0.99% for ischemic heart disease)
Waxman et al., 2018 [32]	Systematic review and meta-analysis (24 studies)	2594, Variable	Carfilzomib	-All-grade CVAE 617 (18.1%) -Grades ≥ 3 CVAE 274 (8.2%) Higher risk with higher doses RR grade ≥3 2.2	NA
Moreau et al., 2018 [33]	ARROW, RCT	578, 12.6 months	Weekly versus twice weekly carfilzomib	Grade ≥3 cardiac failure: -Once weekly group 7 (3%) -Twice weekly group 10 (4%)	NA for cardiac toxicities (total 6.3% AE leading to discontinuation)
Cornell et al., 2019 [37]	PROTECT, Prospective observational	95, 18 months	Carfilzomib or bortezomib	-HF (*n* = 23; 24%) -Grade 3 or 4 Hypertension (*n* = 13;13.6%) AF (*n*=2; 2.1%) Higher risk with carfilzomib	NA
Facon et al., 2019 [52]	CLARION, phase 3 trial	Total 944, 22 months	Carfilzomib or bortezomib in combination with melphalan/prednisone	Grade ≥3: -Cardiac failure 5.1% with carfilzomib and 1.5% with bortezomib -Hypertension 9.1% with carfilzomib and 3% with bortezomib	-Cardiac failure 0.6% with carfilzomib and 0.2% with bortezomib -AF 0.4% with carfilzomib and 0.2% with bortezomib -Hypertesnion 0.8% with carfilzomib and 0% with bortezomib
Bishnoi et al., 2020 [56]	SEER Medicare, Real world	815 (vs 6515 control)	Carfilzomib regimens	-HR 1.47; *p*<0.001 for HF -HR 1.45; *p*<0.001 for ischemic heart disease -HR 3.33; *p*<0.0001 for hypertension	NA
Dimopoulos et al., 2020 [38]	CANDOR, Phase 3 randomized trial	466, 17.2 months	Daratumumab with carfilzomib and dexamethasone versus carfilzomib with dexamethasone	-Cardiac failure around 8% (*n*=39) total Higher with carfilzomib and dexamethasone vs triplet -Ischemic heart disease around 3.8% (*n*=18) total	Around 2.4% total related to cardiac failure
Rahman et al., 2021 [29]	Meta-analysis (4 RCTs)	Total 2954, Variable	Carfilzomib-based therapy	HF 8.1% compared to 3.4% in control arm Pooled RR 2.34, 95% CI: 1.66–3.32	1.5% related to cardiac failure
Richardson et al., 2021 [57]	TOURMALINE-MM1, phase III RCT	722 total (360 in ixazomib arm), 85 months	Rd with/out Ixazomib	-Hypertension 6% with ixazomib vs 5% -HF 4% in both arms -Arrhythmias 16% with ixazomib vs 15%	-NA for cardiac toxicity −38.8% for any AE in ixazomib arm
Moreau et al., 2021 [41]	IKEMA, Prospective randomized trial	302 (including 123 controls), 20.7 months	Carfilzomib with dexamethasone with or without isatuximab	-Cardiac failure 7% -Ischemic heart disease 4–5%	NA (8% total AEs with Isatuximab)
Das et al., 2022 [19]	Meta-analysis (20 RCTs)	Total 10373, 23.1–30.7 months	IMiDs or PI	-IMiDs had higher CTACE high-grade (≥grade 3) cardiotoxic events (OR 2.05–95% CI 1.30–3.26) -PI CTACE high-grade (≥grade 3) cardiotoxic events OR 1.67–95% CI (1.17–2.40)-specifically with carfilzomib OR 2.68–95% CI (1.63–4.40)	NA

*RCT* randomized controlled trial, *KRd* carfilzomib, lenalidomide, dexamethasone, *Rd* lenalidomide, dexamethasone, *MI* myocardial infarction, *NA* not available, *VD* ventricular dysfunction, *HR* hazard ratio, *CI* confidence interval, *HF* heart failure, *CVAE* cardiovascular adverse events, *RR* relative risk, *AE* adverse effects, *AF* atrial fibrillation, *SEER* surveillance, epidemiology and end results, *IMiDs* immunomodulator agents, *PI* proteasome inhibitor, *CTCAE* common terminology criteria for adverse events, *OR* odds ratio.

## Cardiovascular risk assessment, monitoring and management

It is important to assess baseline CV risk factors to identify those at higher risk for cardiac complications. The patient’s age, comorbidities, clinical examination, in addition to specific cardiac function markers should be documented before initiation of treatments that are associated with high risk for cardiac toxicity. Similar to other malignancies, a comprehensive geriatric assessment (CGA), utilizing a multidisciplinary diagnostic; as well as management plans for elderly patients with MM would allow the identification of high-risk individuals who might require dose adjustments and would benefit from regular assessment for any cardiotoxicity. This evaluation should include age, functional status, associated medical conditions, evaluation of polypharmacy and expected drug interaction among others [49]. Multiple assessment tools have been evaluated and incorporated in this comprehensive assessment including the mini-nutritional assessment short form (MNA-SF), Geriatric Depression Scale (GDS), the Mini-Mental State Examination (MMSE), quality of life questionnaire (SF-36), and the Charlson Comorbidity Index (CCI), among others [49–51]. Yao et al. recently noted that around 43.3% of patients with newly diagnosed MM were categorized as frail as per the International Myeloma Working Group (IMWG-GA) index, where even many of those labeled as fit were found to be at risk for malnutrition (43.5%) and depression (24.3%) [50]. The European Society of Cardiology (ESC) position paper set out the proposed tools for evaluation of cardiotoxicity during treatment [4]. There is the readily available electrocardiogram (ECG), and transthoracic echocardiography (TTE) which avoids radiation (evaluating LVEF/GLS) which can predict and detect cardiac dysfunction. There are additionally more advanced imaging techniques that have high reproducibility (radionuclide angiography with multigated radionuclide angiography [MUGA] scans, and cardiac magnetic resonance imaging [MRI]), but these may not be commonly available, or if they are, there may be a waiting list. The QT interval and associated risk factors for QT prolongation should be assessed before; as well as during treatment. Electrocardiograms should be carried out on a regular basis, depending on treatment regimen. Risk factors for QT prolongation such as electrolyte imbalance, (related to nausea and emesis, diarrhea, treatment with loop diuretics among others), hypothyroidism, concurrent use of QT-prolonging drugs *e.g*., antiarrhythmic, anti-microbial agents, psychotropic, antidepressant, antipsychotic, antiemetic, antihistamine should be identified and corrected when possible. Non-modifiable risk factors include family history of sudden death, personal history of syncope, baseline QTc interval prolongation, female gender, advanced age, heart disease, MI, impaired renal function, impaired hepatic drug metabolism among others [4], and these should be taken into consideration when deciding on treatment regimens as well as monitoring intervals. For particular patients there is value to having cardiac biomarker tests (TroponinI, high-sensitivity Troponin I, BNP, N-type terminal fragment BNP [NT-proBNP]), which can be measured at different time points before and during treatment. Those are widely available, accurate, reproducible and have a high sensitivity, however their role in routine surveillance has not been clearly established [4].

**Fig. 2:**
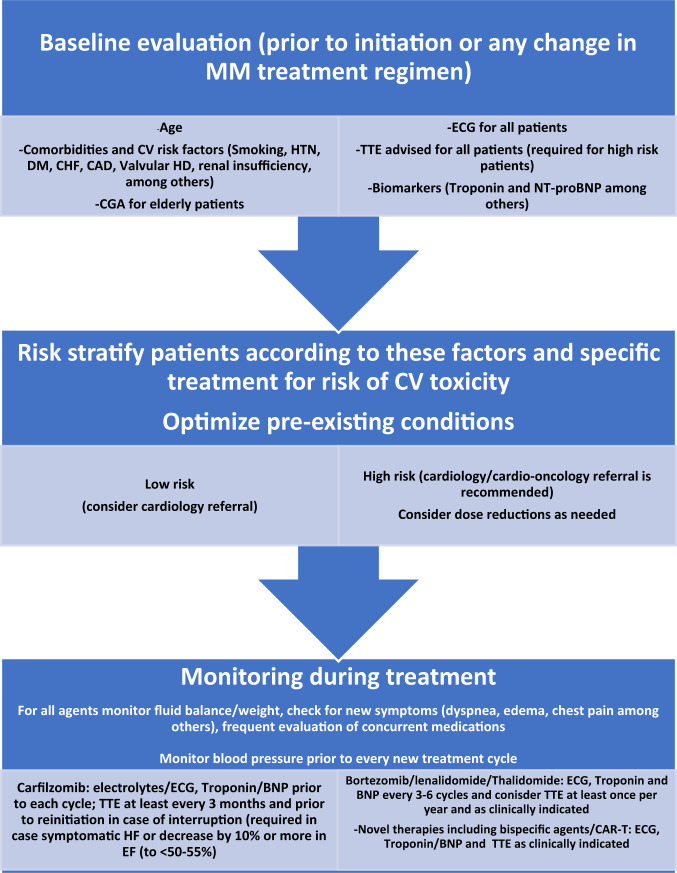
Suggested cardiac monitoring prior to and during Multiple myeloma treatment. MM multiple myeloma, CV cardiovascular, HTN hypertension, DM diabetes mellitus, CHF congestive heart failure, CAD coronary artery disease, HD heart disease, CGA comprehensive geriatric assessment, ECG electrocardiogram, TTE transthoracic echocardiogram, NT-proBNP N-terminal pro-B-type natriuretic peptide, EF ejection fraction, CAR-T chimeric antigen receptor T-cell therapy.

If the patient is at high-risk or the chosen therapy is associated with an increased CV risk, then a cardiologist or cardio-oncologist referral is recommended [7]. An European Society for Medical Oncology (ESMO) consensus article has proposed a monitoring and management approach for patients undergoing potentially cardiotoxic anticancer therapy, incorporating not just a one-time evaluation but a continuous and recurrent reassessment throughout treatment, where patients must be carefully followed so that if there is a suspicion of cardiac failure or other cardiac events, appropriate measures would be undertaken and acted upon accordingly. The incorporation of surveillance strategies in cancer survivors would help prevent the potential long-term CV morbidity and mortality associated with oncological treatments [4]. The lack of the availability or implementation of specific international, national, and/or institutional consensus guidelines for patients with MM regarding the frequency and interpretation of many of the monitoring tools, is one of the factors prohibiting optimal patient care for this population. Figure 2 includes the proposed monitoring and serial testing during different treatments; as well as the need for referral to cardiologists/cardio-oncologists and/or treatment interruption/discontinuation based on available data.

Prevention remains a key to improve patient care in this setting. There are some cardioprotective drugs that we can give in addition to specific thromboprophylaxis whenever this is needed. There are a small number of studies to suggest that angiotensin-converting enzyme (ACE) inhibitors such as enalapril, angiotensin receptor blockers (ARBs) such as candesartan, and selected beta blockers (BBs) such as carvedilol and nebivolol, may be the preferred agents to reduce the risk of cardiotoxicity^;^ in addition to mineralocorticoid receptor antagonists (MRAs) such as spironolactone [52].

## Conclusion

A large proportion of MM patients have CV risk factors or can develop them along their treatment/disease course. Anti-myeloma drugs can be associated with specific CV toxicities including HF, arrhythmias, and hypertension. Preventive strategies for CV health are recommended in addition to specific myeloma-related supportive care. In particular, carfilzomib use can be associated with HF and hypertension, requiring preventive measures and vigilance. In case of doubt, it is imperative that a patient be referred to a cardiologist, or cardio-oncologist before and/or during treatment.

## Supplementary information

Related Manuscript File
